# Erythropoietin Attenuates Pulmonary Vascular Remodeling in Experimental Pulmonary Arterial Hypertension through Interplay between Endothelial Progenitor Cells and Heme Oxygenase

**DOI:** 10.3389/fped.2015.00071

**Published:** 2015-08-28

**Authors:** Rosa Laura E. van Loon, Beatrijs Bartelds, Frank A. D. T. G. Wagener, Nada Affara, Saffloer Mohaupt, Hans Wijnberg, Sebastiaan W. C. Pennings, Janny Takens, Rolf M. F. Berger

**Affiliations:** ^1^Center for Congenital Heart Diseases, University Medical Center Groningen, Beatrix Children’s Hospital, University of Groningen, Groningen, Netherlands; ^2^Department of Experimental Cardiology, University Medical Center Groningen, University of Groningen, Groningen, Netherlands; ^3^Department of Orthodontics and Craniofacial Biology, Radboud University Medical Center, Nijmegen, Netherlands

**Keywords:** hemodynamics, pulmonary vascular remodeling, neointimal lesions, aorto-caval shunt, pulmonary hypertension, right ventricular hypertrophy

## Abstract

**Background:**

Pulmonary arterial hypertension (PAH) is a pulmonary vascular disease with a high mortality, characterized by typical angio-proliferative lesions. Erythropoietin (EPO) attenuates pulmonary vascular remodeling in PAH. We postulated that EPO acts through mobilization of endothelial progenitor cells (EPCs) and activation of the cytoprotective enzyme heme oxygenase-1 (HO-1).

**Methods:**

Rats with flow-associated PAH, resembling pediatric PAH, were treated with HO-1 inducer EPO in the presence or absence of the selective HO-activity inhibitor tin-mesoporphyrin (SnMP). HO activity, circulating EPCs and pulmonary vascular lesions were assessed after 3 weeks.

**Results:**

In PAH rats, circulating EPCs were decreased and HO activity was increased compared to control. EPO treatment restored circulating EPCs and improved pulmonary vascular remodeling, as shown by a reduced wall thickness and occlusion rate of the intra-acinar vessels. Inhibition of HO activity with SnMP aggravated PAH. Moreover, SnMP treatment abrogated EPO-induced amelioration of pulmonary vascular remodeling, while surprisingly further increasing circulating EPCs as compared with EPO alone.

**Conclusion:**

In experimental PAH, EPO treatment restored the number of circulating EPCs to control level, improved pulmonary vascular remodeling, and showed important interplay with HO activity. Inhibition of increased HO activity in PAH rats exacerbated progression of pulmonary vascular remodeling, despite the presence of restored number of circulating EPCs. We suggest that both EPO-induced HO-1 and EPCs are promising targets to ameliorate the pulmonary vasculature in PAH.

## Introduction

Pulmonary arterial hypertension (PAH) is a life-threatening pulmonary vascular disease (1), affecting pediatric patients as well as young adults (2). Despite decades of research, pediatric patients with PAH still have a 5-year mortality rate of ~40% (2). One of the problems is that the currently available medication does not target the proliferative vascular lesions that are typical for PAH (3–5), i.e., neointimal and plexiform lesions. These lesions of the small vessel walls are formed by proliferation of endothelial and smooth muscle cells, fibrosis, thrombosis, and inflammation (4). Although the histomorphology of PAH is well described, the cellular mechanisms underlying this process are still unknown (6).

There is increasing evidence that endothelial cell dysfunction and loss of endothelial cells play a central role in the pathogenesis of pulmonary vascular remodeling (7–9). Hence, therapeutic strategies should be aimed at mobilization of endothelial cells for vascular repair (10). One of these strategies is mobilization of bone marrow-derived endothelial progenitor cells (EPCs) (10). Indeed in hypoxic pulmonary hypertension (PH), mobilization of EPC appears beneficial in reducing media hypertrophy (11). However, vascular lesions in hypoxic PH do not represent the vessel remodeling seen in PAH (3) and the effects of EPCs on vascular remodeling in neointimal proliferation are still unknown.

To study the pathogenesis and potential therapeutic strategies, we use a well-characterized animal model of flow-induced PAH, representing patients with PAH associated with congenital heart diseases (5, 6, 12). In this model, we have recently shown that erythropoietin (EPO) improves pulmonary vascular remodeling through unknown mechanisms (13). EPO is a hypoxia-induced hormone that promotes erythropoiesis. Furthermore, EPO has been demonstrated to exert therapeutic effects in various disorders affecting the vasculature, including post mechanical vascular injury (14) and myocardial and hind-limb ischemia (15, 16). These therapeutic effects may be mediated by the mobilization and homing of EPCs from the bone marrow to the injured vascular endothelium, leading to re-endothelialization and neovascularization (11, 15).

Alternatively, induction of heme oxygenase-1 (HO-1) has been implicated to play an important role in EPOs downstream mechanisms (17–19). HO-1 is the inducible isoform of heme oxygenase (HO) which catalyzes the rate-limiting step in the degradation of heme to ferrous iron, carbon monoxide, and biliverdin. Biliverdin is then rapidly converted to the powerful anti-oxidant bilirubin by biliverdin reductase (20). HO-1 is expressed in response to a variety of stresses and, in turn, its reaction products protect against oxidative and inflammatory stress and apoptosis (21, 22). In experimental models of hypoxia-induced PH, characterized by isolated media hypertrophy and not the PAH-specific obliterative pulmonary vascular remodeling, overexpression of HO-1 appears to protect against the development of PH (23, 24).

In the present study, we postulated that the beneficial effects of EPO on pulmonary vascular remodeling in PAH, are through induction of HO activity and mobilization of EPCs. To test this hypothesis, we treated rats with (flow-associated) neointimal PAH with EPO in the presence or absence the potent selective HO-activity inhibitor, tin-mesoporphyrin (SnMP), and measured pulmonary vascular remodeling, hemodynamics, pulmonary HO activity, and the number of circulating EPCs.

## Materials and Methods

### Animals and study design

Seventy-two male Wistar rats (280–300 g) were purchased from Harlan (Zeist, The Netherlands). The experimental protocol was approved by the institutional Animal Care and Use Committee (University Medical Center Groningen). Animal care and experiments were conducted according to the US National Institutes of Health guidelines.

Rats were randomized into five groups: (1) flow-associated PAH untreated (PAH, *n* = 17), (2) PAH treated with EPO (PAH + EPO, *n* = 17), (3) PAH treated with EPO and the HO-inhibitor SnMP (PAH + EPO + SnMP, *n* = 13), (4) PAH treated with only SnMP (PAH + SnMP, *n* = 12), and (5) a control group (CON, *n* = 13). Flow-associated PAH was created by subcutaneous injection with monocrotaline (60 mg/kg, Sigma Chemical Co., St. Louis, MO, USA) at day 0, followed by an abdominal aorto-caval shunt at day 7, as described in detail previously (6). EPO (40 μg/kg, Darbepoetin-alpha, Aranesp^®^, Amgen Inc., Thousand Oaks, CA, USA) was administered via a single subcutaneous injection on the day of aorto-caval shunt surgery as described previously (13, 16). SnMP (30 μmol/kg, Sn(IV) Mesoporphyrin IX dichloride, Frontier Scientific Inc.) was administered via two subcutaneous injections: on the day of aorto-caval shunt surgery and 10 days later. The control group underwent subcutaneous saline injection followed by sham surgery. For analgesia, all rats received buprenorphine (0.1 mg/kg s.c. tid) for 2 days postoperatively. For measurement of hematocrit, blood (200 μL) was drawn every week starting from baseline (*t* = 0, day of monocrotaline injection). For isolation of circulating EPCs, blood (1 mL) was drawn at baseline and before sacrifice (*t* = 28).

Of the 72 rats, all but eight successfully completed the experimental protocol. In four of these eight rats, shunt surgery was not successful: two rats died due to acute surgery-related complications (PAH *n* = 1, PAH + SnMP, *n* = 1), one underwent early elective sacrifice because of postoperative paresis of hind legs (PAH) and in one rat there was no shunt present at sacrifice (PAH). Of the other four rats, one underwent early elective sacrifice because of extensive unexplained wounds to the snout (CON), one died prematurely due to unknown causes (PAH + EPO) and two rats died during orbital function for blood withdrawal (PAH + EPO, *n* = 1; PAH + SnMP, *n* = 1). All these eight rats were excluded from analysis.

### Circulating endothelial progenitor cells

For measuring the levels of circulating EPCs, we used a cell culturing and staining technique that has been described previously (16, 25), since fluorescence activated cell sorting (FACS) is impossible for identifying rat EPCs due to the lack of rat-specific EPC antigens.

Mononuclear cells were isolated from 1 mL of peripheral blood by density gradient centrifugation with Histopaque-1083, according to manufacturer’s instructions (Sigma Chemical, St. Louis, MO, USA). Isolated mononuclear cells were seeded in triplicate on 1% gelatine-coated 96-well plates (BD BioCoat, Bedford, MA, USA), in endothelial cell basal medium (Cambrex Bioproducts, Clonetics, NJ, USA) supplemented with 2% fetal bovine serum, EGM-2 SingleQuots (Clonetics), Penicillin (100 U/mL), and Streptomycin (100 μg/mL). After 2 days of culture, the adherent cells were carefully washed with medium and co-stained with DiI AcLDL (1,1′-dioctadecyl-3,3,3′,3′-tetramethylindocarbocyanine-labeled acetylated Low-Density Lipoprotein, 10 μg/mL; Molecular Probes, Invitrogen, Carlsbad, CA, USA) and FITC-conjugated BS lectin 1 (fluorescein isothyiocyanate-labeled Bandeiraea Simplicifolia lectin 1, 10 μg/mL; Sigma-Aldrich, Zwijndrecht, Netherlands). DAPI (4′,6-diamidino-2-phenyindole, dilactate, Sigma-Aldrich, Zwijndrecht, Netherlands) was used for nuclear staining. Images were captured using an LSM 410 fluorescence microscope with a magnification of 100× (Carl Zeiss, Jena, Germany). Double positive cells for DiI AcLDL and lectin staining were considered an EPC and counted by co-localization analysis using Image-Pro Plus for Windows software (version 4.5) by an investigator blinded to treatment. DAPI staining served as a control to see whether the double positive cells were cells with a nucleus. For every blood sample, the average number of EPCs was calculated from six high-power field images, two from each well.

### Hemodynamic measurements

Three weeks after aorto-caval shunt surgery (*t* = 28), we anesthetized the animals and measured hemodynamics as described previously. Briefly, pulmonary and systemic arterial pressures were measured using fluid-filled catheters and right ventricular contractility was measured using a 2-French Microtip pressure transducer (Millar Instr. Inc., Houston, TX, USA). After completion of hemodynamic measurements, blood samples were drawn from the left carotid artery, distal, and proximal inferior caval vein (below and, respectively, above the level of the aorto-caval shunt) in order to determine oxygen saturation and confirm the presence of a shunt by significant oxygen step-up (31 ± 10%).

### Pulmonary vascular remodeling

Pulmonary morphometric analysis was assessed as described previously (5, 6, 9). Briefly, the right lung was frozen in liquid nitrogen for molecular analyses. The left lung was fixated by filling the airways with 3.6% formalin at a pressure of 20 cmH_2_O. Deparaffinized pulmonary 5 μm thick sections were stained with hematoxylin–eosin and Verhoef elastin-stain for morphometric analysis of vascular dimensions. In the lung sections, all transversally cut arteries with a diameter ≥50 μm (pre-acinar arteries) and 40 randomly chosen vessels (10 in each left lung quadrant) with a diameter <50 μm (intra-acinar vessels) were quantitatively analyzed at 200 and 400× magnification using image analysis software (Image-Pro Plus for Windows, version 4.5). In the pre-acinar arteries, wall thickness and medial wall/lumen ratio were measured and calculated. In the intra-acinar pulmonary vessels, wall thickness was measured in addition to occlusion scores, calculated according to the following formula: (outer vessel area − luminal area)/(outer vessel area). Muscularization of 40 small pulmonary vessels was assessed as described previously (6, 12).

### Heme oxygenase activity measurement

To determine pulmonary HO activity at day 28, we performed an HO-activity assay by measurement of bilirubin (and biliverdin) generation using HPLC techniques, slightly modified from previously described (26). In short, proteins were isolated from frozen lung by pulverization using a Mikro-dismembrator (Sartorius B. Braun Biotech Int., Melsungen, Germany, 2000 rpm for 40 s). Lung homogenate was resuspended (40 mg/mL) in lysis buffer (20 mM Tris/250 mM sucrose, pH = 8.5; supplemented with protease inhibitors) and subsequently lysed by three freeze-thaw cycles. Next, the lysate was centrifuged at 13,000 rpm at 4°C for 10 min. The supernatant was used for the HO-activity assay and the measured protein concentration was used for normalization. Samples for measurement on an HPLC (Spectra-Physics Analytical, Spectrasystem SCM400) equipped with a 5 μm Discovery C18 column and a Discovery C18 Supelguard Cartridge 5 μm particle size precolumn (both Sigma-Aldrich) were supplemented with vitamin C to prevent oxidation of bilirubin or biliverdin. As controls for HO activity, we used untreated fibroblasts in combination with fibroblasts treated for 24 h with 10 μM curcumin (26).

### Quantitative real-time-PCR

Real-time PCR (RT-PCR) was performed using standard assays. Total RNA was isolated from pulmonary tissue using TRIzol reagent (Invitrogen). RT-PCR experiments were performed on a CXF384 real-time system C1000 Thermal cycler (BioRad Laboratories, Veenendaal, The Netherlands). cDNA was synthesized using Moloney murine leukemia virus reverse transcriptase and random primers (Invitrogen, Burlington, ON, Canada). RT-PCR was conducted using SYBR Green PCR Master Mix according to the manufacturer’s instructions (Eurogentec, San Diego, CA, USA). Primer sequences are available on request. Expression levels were obtained from a dilution standard curve and compared with those of 36B4 RNA in order to calculate the relative expression levels.

### Statistical analysis

Data are presented as mean ± SEM or median (range). Differences between the PAH and CON groups were analyzed using *t*-tests for normally distributed data and Mann–Whitney *U* testing for not normally distributed data. Differences between the untreated and treated PAH groups (groups 1–4) were tested by one-way ANOVA followed by Fisher’s protected LSD *post hoc* testing (hematocrit, hemodynamics, RV hypertrophy, and pulmonary vascular remodeling) and Kruskall–Wallis followed by Mann–Whitney *post hoc* testing with Bonferroni correction (number of EPCs) was used. Alpha was chosen to be 0.05. A two-tailed *p*-value <0.05 was considered statistically significant. SPSS 16.0 was used to perform the analyses.

## Results

### Flow-associated PAH rat model

**Figure 1:**
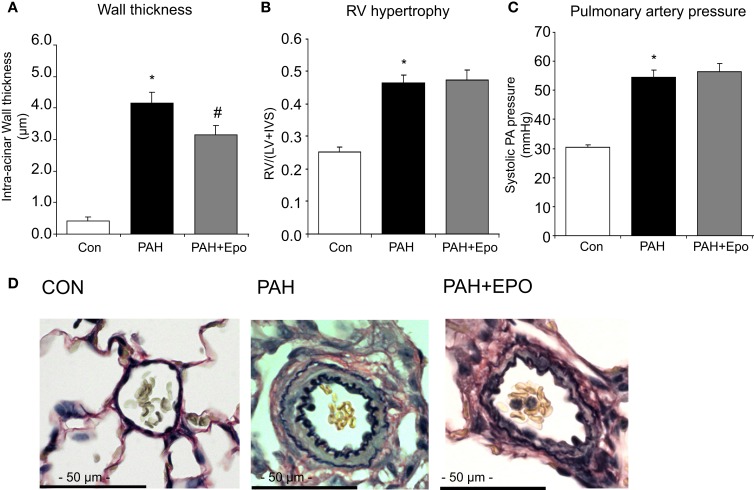
**Flow-associated PAH and effects of EPO treatment**. Flow-associated PAH and effects of EPO treatment on **(A)** wall thickness of intra-acinar pulmonary vessels, **(B)** right ventricular hypertrophy [RV/(LV + IVS)], and **(C)** systolic pulmonary arterial pressure. **(D)** Examples of pulmonary histopathology (Verhoeff-staining) of intra-acinar pulmonary vessels*. CON*, control group, *n* = 12; *PAH*, untreated pulmonary arterial hypertension, *n* = 12–14; *PAH* + *EPO*, PAH treated with erythropoietin (EPO), *n* = 12–15. Differences between the PAH and CON groups were analyzed using *t*-tests for normally distributed data and Mann–Whitney *U* testing for not normally distributed data. Differences between the untreated and treated PAH groups (groups 1–4) were tested by one-way ANOVA followed by Fisher’s protected LSD *post hoc* testing (hematocrit, hemodynamics, RV hypertrophy, pulmonary vascular remodeling). *p < 0.05 vs. CON, ^#^*p* < 0.05 vs. PAH.

Flow-associated PAH was confirmed by histology, pathology, and hemodynamics as described previously (5, 6). Briefly, PAH rats showed advanced pulmonary vascular remodeling, including significantly increased wall thickness, occlusion scores and muscularization of intra-acinar vessels as well as increased wall thickness and wall/lumen ratio of pre-acinar pulmonary vessels as compared to control (Figures 1A,D; Table 1). PAH rats had significantly increased right ventricular hypertrophy, increased right atrial and pulmonary arterial pressures, and decreased right ventricular contractility (Figures 1B,C; Table 1).

**Table 1:** **Animal characteristics**.

	CON	PAH	PAH + EPO	PAH + EPO + SnMP	PAH + SnMP
**Hemodynamics**
Mean pulmonary artery pressure	20 ± 1	32 ± 1*	35 ± 1	37 ± 3	34 ± 2
Systolic pulmonary arterial pressure (mmHg)	30 ± 1	54 ± 3*	56 ± 3	56 ± 4	56 ± 6
Mean systemic arterial pressure (mmHg)	89 ± 2	63 ± 4*	71 ± 6	71 ± 5	67 ± 4
dP/dT indexed max	106 ± 8	71 ± 2*	76 ± 6	74 ± 7	67 ± 6
−dP/dT indexed max	91 ± 7	55 ± 16*	80 ± 6	56 ± 18	62 ± 7
Heart rate (beats per minute)	343 ± 20	310 ± 14	321 ± 15	312 ± 18	328 ± 13
**Pathology**
Body weight at sacrifice (g)	390 ± 8	350 ± 8*	346 ± 5	343 ± 5	343 ± 5
RV hypertrophy, RV/(LV + IVS)	0.25 ± 0.01	0.47 ± 0.03*	0.47 ± 0.03	0.47 ± 0.03	0.42 ± 0.04
RV weight (mg)	0.21 ± 0.02	0.44 ± 0.02	0.44 ± 0.03	0.40 ± 0.03	0.38 ± 0.03
**Pulmonary vascular morphometry**
**Intra-acinar pulmonary vessels <50 μm**
Totally muscularized	2.6 ± 1.2	20.3 ± 3.5*	18.9 ± 2.6	24.0 ± 5.6	28.8 ± 6.1
Partly muscularized	2.7 ± 0.9	9.4 ± 2.5*	9.6 ± 2.0	17.6 ± 2.6	13.1 ± 3.0
**Pre-acinar pulmonary vessels >50 μm**
Wall thickness (μm)	7.3 ± 1.3	15.6 ± 1.5*	16.1 ± 1.8	15.4 ± 1.9	16.6 ± 3.1
Wall/lumen ratio	0.08 ± 0.01	0.15 ± 0.01*	0.14 ± 0.01	0.15 ± 0.02	0.24 ± 0.05^§^^,^^#^

*Data are presented as mean ± SEM. *CON*, control group, *n* = 12; *PAH*, untreated pulmonary arterial hypertension, *n* = 14; *PAH* + *EPO*, PAH treated with erythropoietin (EPO), *n* = 15; *PAH* + *EPO* + *SnMP*, PAH treated with EPO and tin-mesoporphyrin (SnMP), *n* = 13; *PAH* + *SnMP*, PAH treated with SnMP, *n* = 10; *RV*, right ventricle; *(−)dP/dT indexed max*, RV contractility, maximal rate of increase or decrease in RV pressure [calculated as the variation in pressure (*P*) with time (*T*) corrected for RV systolic pressure]; *LV*, left ventricle; *IVS*, interventricular septum. Differences between the PAH and CON groups were analyzed using *t*-tests for normally distributed data and Mann–Whitney *U* testing for not normally distributed data. Differences between the untreated and treated PAH groups (groups 1–4) were tested by one-way ANOVA followed by Fisher’s protected LSD *post hoc* testing (hematocrit, hemodynamics, RV hypertrophy, pulmonary vascular remodeling)*.

*p < 0.05 vs. CON.

*^#^*p* < 0.05 vs. PAH*.

*^§^*p* < 0.05 vs. PAH + EPO + SnMP*.

### Increased HO activity and decreased number of circulating EPCs in PAH

**Figure 2:**
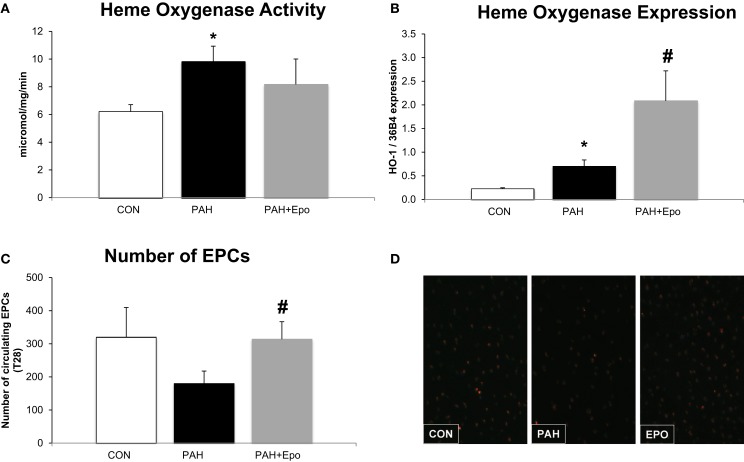
**Effect of EPO treatment on HO activity and EPCs**. **(A)** Heme oxygenase activity measured in whole lung lysates (methods). **(B)** Heme oxygenase mRNA expression, relative to 36B4 expression in whole lung. **(C)** Number of Endothelial Progenitor Cells (EPC) in blood at the day of sacrifice. **(D)** Example of fluorescence microscope picture of EPCs in blood. *CON*, control group, *n* = 4–8; *PAH*, untreated pulmonary arterial hypertension, *n* = 8–12; *PAH* + *EPO*, PAH treated with erythropoietin (EPO), *n* = 5–8. Differences between the PAH and CON groups were analyzed using *t*-tests for normally distributed data and Mann–Whitney *U* testing for not normally distributed data. Differences between the untreated and treated PAH groups (groups 1–4) were tested by one-way ANOVA followed by Fisher’s protected LSD *post hoc* testing (hematocrit, hemodynamics, RV hypertrophy, pulmonary vascular remodeling) and Kruskall–Wallis followed by Mann–Whitney *post hoc* testing with Bonferroni correction (number of EPCs) was used. *p < 0.05 vs. CON, ^#^*p* < 0.05 vs. PAH.

At day 28, rats with PAH had increased pulmonary HO activity and HO-1 mRNA levels (Figures 2A,B). The number of circulating EPCs tended to be lower in PAH, although not reaching statistical significance (*p* = 0.1, Figure 2C). There were no differences in baseline EPC number or hematocrit (data not shown).

### Effects of EPO in PAH

**Figure 3:**
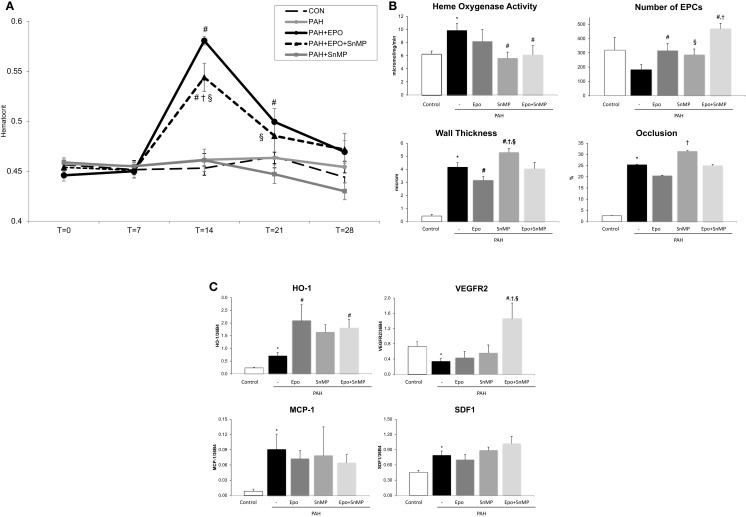
**(A)** Hematocrit levels throughout the study time points at *x*-axis in days. Data are mean ± SEM. *CON*, control group, *n* = 10; *PAH*, untreated pulmonary arterial hypertension, *n* = 12; *PAH* + *EPO*, PAH treated with erythropoietin (EPO), *n* = 14. *p < 0.05 vs. CON, ^#^*p* < 0.05 vs. PAH *PAH* + *EPO* + *SnMP*, PAH treated with EPO and tin-mesoporphyrin (SnMP), *n* = 12; *PAH* + *SnMP*, PAH treated with SnMP, *n* = 9. **(B)** Effects of HO activity blockade on HO activity, number of circulating EPCs and pulmonary vascular remodeling. *CON*, control group, *n* = 4–12; *PAH*, untreated pulmonary arterial hypertension, *n* = 8–14; *PAH* + *EPO*, PAH treated with erythropoietin (EPO), *n* = 5–15. *PAH* + *EPO* + *SnMP*, PAH treated with EPO and tin-mesoporphyrin (SnMP), *n* = 5–13; *PAH* + *SnMP*, PAH treated with SnMP, *n* = 6–10. **(C)** Effects of HO activity blockade gene expression. *CON*, control group, *n* = 5; *PAH*, untreated pulmonary arterial hypertension, *n* = 6; *PAH* + *EPO*, PAH treated with erythropoietin (EPO), *n* = 6. *PAH* + *EPO* + *SnMP*, PAH treated with EPO and tin-mesoporphyrin (SnMP), *n* = 6; *PAH* + *SnMP*, PAH treated with SnMP, *n* = 6.*VEGF-R2*, vascular endothelial growth factor receptor 2; *SDF-1*, stromal derived growth factor-1; *MCP-1*, monocyte chemoattractant protein-1. mRNA expression levels relative to 36B4 mRNA levels. Differences between the PAH and CON groups were analyzed using *t*-tests for normally distributed data and Mann–Whitney *U* testing for not normally distributed data. Differences between the untreated and treated PAH groups (groups 1–4) were tested by one-way ANOVA followed by Fisher’s protected LSD *post hoc* testing (hematocrit, hemodynamics, RV hypertrophy, and pulmonary vascular remodeling) and KruskallâĂŞWallis followed by MannâĂŞWhitney post hoc testing with Bonferroni correction (number of EPCs) was used. ^#^*p* < 0.05 vs. PAH, ^†^*p* < 0.05 vs. PAH + EPO, ^§^*p* < 0.05 vs. PAH + SnMP.

EPO treatment improved pulmonary vascular remodeling, as previously shown by reduced wall thickness and occlusion scores of intra-acinar pulmonary vessels, compared with PAH (Figures 1A,D; Table 1). Hemodynamics remained unchanged, which is consistent with previous experiments (13). EPO treatment did not further increase pulmonary HO activity at day 28 (Figure 2A), whereas HO-1 mRNA expression levels were further increased (Figure 2B). EPO treatment increased the number of circulating EPCs to levels similar to CON (Figure 2C). Hematocrit levels increased significantly after EPO treatment (Figure 3A) demonstrating the successful administration of EPO. Taken together, EPO improved pulmonary vascular remodeling and increased the number of circulating EPCs.

### Effects of inhibition of HO activity in PAH

SnMP successfully inhibited HO activity in both PAH + SnMP and PAH + EPO + SnMP to levels as measured in CON (Figure 3B), whereas SnMP did not decrease HO-1 mRNA levels (Figure 3C), which confirms its function in inhibiting HO activity and not HO-1 transcription. SnMP treatment in the absence of EPO treatment aggravated pulmonary vascular remodeling to levels worse than seen in untreated PAH: significantly increased wall thickness and occlusion scores of intra-acinar pulmonary vessels and increased wall-lumen ratio of pre-acinar pulmonary arteries (Figure 3B; Table 1). Interestingly, SnMP treatment alone tended to increase circulating EPCs (PAH + SnMP vs. PAH *p* = 0.09) to a level comparable to that after EPO treatment (PAH + EPO; Figure 3B). Taken together, inhibition of HO activity worsened pulmonary vascular remodeling, despite increasing the number of circulating EPCs.

### Effects of EPO and inhibition of HO activity in PAH

EPO-mediated amelioration of pulmonary vascular remodeling was abrogated following co-treatment with SnMP, while further increasing the number of circulating EPCs compared to EPO treatment alone (Figure 3B). EPO-induced HO-1 mRNA levels remained high in the presence of the HO-activity inhibitor SnMP (Figure 3C). Taken together, inhibition of HO activity strongly attenuated the protective effects of EPO on pulmonary vascular remodeling despite further increasing the number of circulating EPCs.

### Effects of EPO and inhibition of HO activity on EPC homing factors and markers of inflammation

In order to investigate the effects of EPO on factors involved in the homing of circulating EPCs to the diseased vessel endothelium, we studied two stimuli required for EPCs to home to diseased vascular walls: vascular endothelial growth factor (VEGF) and its two receptor subtypes (VEGF-R1, VEGF-R2) and stromal derived growth factor-1 (SDF-1) (16, 27). Since inflammatory processes are involved in the pathophysiology of PAH and since HO activity has potent anti-inflammatory properties (28), we also measured monocyte chemo attractant protein-1 (MCP-1) levels, a major inflammatory marker, in order to study the effects of modulation of HO activity using EPO and SnMP on inflammatory processes in PAH.

Pulmonary VEGF-R2 mRNA expression levels were decreased and SDF-1 levels were increased in PAH. After EPO treatment, these mRNA levels were unchanged (Figure 3C). Interestingly, EPO treatment together with SnMP tended to increase mRNA expression levels of VEGF-R2 and SDF-1, whereas SnMP alone did not alter these mRNA levels. Regarding inflammation, MCP-1 mRNA expression levels were significantly increased in PAH rats, compared to CON (Figure 3C). EPO treatment with or without SnMP did not influence these levels. Taken together, EPO and SnMP did not influence EPC homing factors or markers of inflammation.

## Discussion

EPO treatment has been demonstrated to have beneficial effects on pulmonary vascular remodeling in PH. This study describes the interplay between EPO treatment, circulating EPCs and HO activity in a rat model of experimental flow-associated PAH. This model is characterized by a neointimal-type of pulmonary vascular remodeling, a decreased number of circulating EPCs, while both gene expression and activity of HO-1 are increased; Treatment with EPO restored the number of circulating EPCs and was associated with improved vascular remodeling. Twenty-one days after EPO administration, no change in HO activity could be demonstrated, although HO-1 gene expression was further increased. In contrast, reducing HO-1 activity to control level using tin-mesoporphyrin seriously aggrevated pulmonary vascular remodeling, despite an associated increase in circulating EPCs. The combination of EPO treatment and simultaneously reduced of HO activity (to control level) resulted in a cumulative increase in circulating EPCs, however without any improvement of pulmonary vascular remodeling.

What do we learn from these observations regarding the mechanisms by which EPO treatment exerts its ameliorating effects on pulmonary vascular remodeling in PAH?

### Endothelial progenitor cells

Consistent with previous experimental data and with recent reports in human PAH, both idiopathic and associated with congenital heart disease, we observed decreased number of circulating EPCs in untreated PAH (13, 29, 30). This shortage of circulating EPCs in PAH may contribute to the progression of pulmonary endothelial dysfunction and pulmonary vascular remodeling, since EPCs are believed to be able to incorporate into damaged endothelium and potentially repair this endothelium. Bone marrow-derived EPCs have been observed in diseased pulmonary vessel walls of experimental models of untreated hypoxia and monocrotaline-induced PH (31, 32) and the incorporation of EPCs into this diseased pulmonary endothelium has been suggested to improve pulmonary vascular remodeling (11). Until now, such improvements have been observed only after administration of genetically modified and functionally enhanced EPCs in models of media hypertrophy type of pulmonary vascular remodeling (33, 34).

The role of bone marrow-derived progenitor cells and their tissue-restoring potential in PAH remain largely unknown and whether the beneficial effect of EPO treatment in PAH is through this mechanism remains to be demonstrated. In the current study, reducing HO activity by SnMP in experimental PAH, induced an increase in circulating EPCs, but was associated with a further deterioration of pulmonary vascular remodeling. This observation implies that HO activity is crucially important in PAH, either for circulating EPCs to exert their healing effect on the pulmonary vasculature, or because of an EPC-independent vascular protective HO-effect, for which the increase in EPCs could not compensate. In both explanations, HO-1 appears crucial for a vascular protective effect in PAH.

Available evidence supporting the first concept that HO-1 activity is essential for EPCs to function normally and to exert the beneficial effect of EPO in PAH includes previous findings that (1) the function of EPCs and its homing to the damaged pulmonary vasculature is hampered in humans with PAH (29, 30); (2) inhibition of HO activity leads to dysfunctional EPCs (35); (3) HO activity promotes EPCs to home (36, 37); (4) beneficial effects of EPO fade out in the presence of HO-activity inhibitors in WT mice and are absent HO-1 knock out mice (17, 19); and (5) HO-1/carbon monoxide enhances re-endothelialization after vascular injury via promoting mobilization of circulating EPCs (38).

### Heme oxygenase activity

It is currently well established that HO-1 expression provides a general cyto- and tissue-protective effect, as a generalized protective response to environmental stressors, including vascular shear stress. These protective effects of HO-1 are contributed to its properties to inhibit or modulate inflammatory, apoptotic and proliferative processes. HO-1 plays an essential role in such processes, via the production of the gasotransmitter carbon monoxide, the anti-oxidant bilirubin, and of iron, that co-induces the iron scavenger ferritin (21, 22). Inflammatory, apoptotic, and proliferative processes have been demonstrated to contribute to the progression of PAH (12, 28) and experimental induction of HO-1, either chemically or using transgenic mice with lung-specific HO-1-overexpression, inhibited the development of PAH in animal models (23, 24). In the present study, HO-1 mRNA and HO-1 activity levels were augmented in untreated PAH and inhibition of HO activity worsened pulmonary vascular remodeling in the presence of normal level of circulating EPCs. This observation supports the concept that increased HO activity reflects an intrinsic protective response to increased inflammatory stress in PAH (39). Indeed, in untreated PAH rats, we found signs of increased inflammation involving highly increased levels of MCP-1, a monocyte chemo attractant and marker for inflammation, analogous to studies in humans with PAH (40). The importance of HO-1 induction in the pulmonary vascular bed is further emphasized by the recent finding that overexpression of HO-1 improves the beneficial effects of mesenchymal stromal cells in hypoxic PH (41). Putatively, HO-1 activation by specific microRNAs may initiate the anti-inflammatory effects, as has been suggested by recent studies in ECs (42).

EPO is known to induce HO activity both in physiological and diseased states. While EPO treatment resulted in a further increase of HO-1 mRNA levels in concordance with previous findings in experimental models of kidney failure and myocardial infarction (17, 19), HO-1 activity, a more specific assessment for functional properties, was not further enhanced. The latter, however, may be explained by the timing of the experiments: EPO-mediated HO-1 induction is likely highest within the first week. After 2 weeks, most of the induced HO-1 activity may have faded. Consequently, the presented HO-activity data may be an underestimation of the initial HO-1 induction by EPO. Interestingly, other potent activators of HO-1, such as the anti-oxidant Protandim, are also successful in increasing the protective response in PAH (43). Enhancement of HO-1 may not only target the pulmonary vascular bed but also the right ventricle, which affects outcome in PAH. Protandim has been shown to target RV fibrosis (44), which is an important feature in RV dysfunction in response to increased afterload (45–48).

Recently, several studies have suggested that HO-1 and its byproduct CO also may induce cytoprotective properties by other mechanisms, then attenuating apoptosis and inflammation. HO-1 potentially impacts the regulation of autophagy, a vital cellular process, which may in part contribute to an independent cytoprotective effect of HO-1 (49).

The results of the current study show that both EPO and HO-1 have beneficial effects on pulmonary vascular remodeling in experimental PAH and both interact with circulating endothelial cells. The results further indicate that all three, EPO, EPCs, and HO-1, are potential therapeutic targets to improve pulmonary vascular remodeling in PAH. Whether intrinsic functioning and/or homing of these circulating EPCs in the pulmonary vasculature is affected by EPO or HO-1 or both cannot be concluded from the current data. Our finding that inhibition of HO activity by SnMP boosted the mobilization of EPCs is intriguing, and could be related to feedback mechanisms that sense the lack of functional EPCs homing to the vasculature or sense the need for clinical improvement. Future experiments to address this question may use chimeric PAH rats, lethally radiated and transplanted with green fluorescent or otherwise marked bone marrow cells, to study the migration and homing of bone marrow-derived EPCs in the pulmonary endothelium and its effects on pulmonary vascular remodeling.

Since HO activity appears to be necessary for EPC-mediated repair, this suggests that EPC therapy for PAH patients could be coupled to HO-1 induction using, e.g., EPO. This is further supported by recent evidence that EPC therapy is more efficient when HIF-1alpha, an inductor of HO-1-expression, is activated (50).

Several limitations should be addressed as they may have influenced the outcome of the study. At first, the design of the present study did not allow us to analyze the incorporation of EPCs within the pulmonary vascular bed, and it is therefore not possible to assess the fate of the increased number of circulating EPCs after EPO and/or HO-inhibition. The isolated mononuclear cells in our study likely contain a mixture of progenitor cells in different developing stages; nevertheless, the majority of them are expected to be EPCs. In this study, we did not use a control group treated with SnMP alone since tissue HO-1 expression is generally low in the absence of stress and SnMP is not causing harm under these conditions. In fact, it has been given as drug to prevent baby jaundice: the uses of synthetic heme analogs (SnMP) that are competitive inhibitors of heme oxygenase, the rate-limiting enzyme in the production of bilirubin, represent a novel means of controlling severe hyperbilirubinemia in the newborn (51). This, and the observation that in the current experiment, SnMP reduced HO-1 activity to levels not lower than controls, makes clear that theoretical HO-1-depletion effects are not to be expected. Also, we used SnMP, which inhibits the activity of both HO-1 and HO-2 isoforms. Hence it is possible that some of the potent effects of SnMP could be attributed in part to inhibition of HO-2 activity. However, HO-2 is the constitutive isoform, whereas HO-1 is the inducible isoform. From the measured mRNA expression levels, one might have concluded that HO-1 was activated upon EPO treatment, but, as mentioned above, in this study we measured the actual HO activity only after 3 weeks, which is likely an underestimation of the HO activity shortly following EPO treatment. Furthermore, the effect of multiple doses of EPO was not addressed in the present study.

In conclusion, in this *in vivo* model of flow-associated PAH we showed that EPO treatment restored the number of circulating EPCs to control level, improved pulmonary vascular remodeling, and showed important interplay with HO activity. Inhibition of increased HO activity in PAH rats exacerbated progression of pulmonary vascular remodeling, despite the presence of restored number of circulating EPCs. Moreover, EPO-induced amelioration of disease was abrogated when HO activity was reduced. These findings suggest that EPO-mediated HO-1 and EPCs both provide potential therapeutic targets in this still devastating pulmonary vascular disease.

## Author Contributions

RL, BB, FW, and RB contributed to the conception and design of the work, to the drafting of the work, or revising it critically for important intellectual content. RL, BB, NA, SM, HW, SP, and JT contributed to the acquisition, analysis, or interpretation of data for the work. RL, BB, FW, NA, SM, HW, SP, JT, and RB gave final approval of the version to be published and agreed to be accountable for all aspects of the work and ensured that questions related to the accuracy or integrity of any part of the work are appropriately investigated and resolved.

## Conflict of Interest Statement

Rosa Laura E. van Loon, Beatrijs Bartelds, Frank A. D. T. G. Wagener, Nada Affara, Saffloer Mohaupt, Hans Wijnberg, Sebastiaan W. C. Pennings, and Janny Takens have no conflict of interest to declare. Rolf M. F. Berger has performed consultancy activities for Actelion Pharmaceuticals, GlaxoSmithKline, Lilly, and United Therapeutics, and has received lecture fees from Actelion Pharmaceuticals. His institution has received grants from Actelion Pharmaceuticals and Pfizer. The Guest Associate Editor Maurice Beghetti declares that, despite having collaborated with author Rolf M. F. Berger, the review process was handled objectively and no conflict of interest exists.
